# Periodicity and Spectral Composition of Light in the Regulation of Hypocotyl Elongation of Sunflower Seedlings

**DOI:** 10.3390/plants11151982

**Published:** 2022-07-29

**Authors:** Dragan Vinterhalter, Branka Vinterhalter, Vaclav Motyka

**Affiliations:** 1 Institute for Biological Research “Siniša Stanković”, National Institute of Republic of Serbia, University of Belgrade, Bulevar Despota Stefana 142, 11060 Belgrade, Serbia; 2 Institute of Experimental Botany of the Czech Academy of Sciences, Rozvojová 263, 165 02 Prague, Czech Republic; motyka@ueb.cas.cz

**Keywords:** diurnal photoperiods, free-running photoperiods, monochromatic LED light, rhythmicity of hypocotyl elongation, light entrainment

## Abstract

This study presents the hypocotyl elongation of sunflower seedlings germinated under different light conditions. Elongation was rhythmic under diurnal (LD) photoperiods but uniform (arrhythmic) under free-running conditions of white light (LL) or darkness (DD). On the sixth day after the onset of germination, seedlings were entrained in all diurnal photoperiods. Their hypocotyl elongation was dual, showing different kinetics in daytime and nighttime periods. The daytime elongation peak was around midday and 1–2 h after dusk in the nighttime. Plantlets compensated for the differences in the daytime and nighttime durations and exhibited similar overall elongation rates, centered around the uniform elongation in LL conditions. Thus, plants from diurnal photoperiods and LL could be grouped together as white-light treatments that suppressed hypocotyl elongation. Hypocotyl elongation was significantly higher under DD than under white-light photoperiods. In continuous monochromatic blue, yellow, green, or red light, hypocotyl elongation was also uniform and very high. The treatments with monochromatic light and DD had similar overall elongation rates; thus, they could be grouped together. Compared with white light, monochromatic light promoted hypocotyl elongation. Suppression of hypocotyl elongation and rhythmicity reappeared in some combination with two or more monochromatic light colors. The presence of red light was obligatory for this suppression. Plantlets entrained in diurnal photoperiods readily slipped from rhythmic into uniform elongation if they encountered any kind of free-running conditions. These transitions occurred whenever the anticipated duration of daytime or nighttime was extended more than expected, or when plantlets were exposed to constant monochromatic light. This study revealed significant differences in the development of sunflower plantlets illuminated with monochromatic or white light.

## 1. Introduction

Hypocotyl elongation is an early developmental process of seedlings that serves to bring the embryo plumule above the soil surface, enabling further autotrophic growth in full daylight. This process has been studied extensively in dicotyledonous plants. Hypocotyl elongation is influenced by numerous factors that can be studied under controlled laboratory conditions. Among the environmental factors, light is the most important [1], as it affects hypocotyl elongation through its intensity, spectral composition, and periodicity [2,3,4]. On the other hand, seedlings are well equipped to receive light signals using a variety of different receptor pigments [5], and they respond by selecting a developmental program suited for the current light conditions. Light provides plants with complex information about conditions characterizing their environment. Plants can detect light direction, intensity, duration, and quality, as well as receive precious information about the passage of time. Time tracking resulting in entrainment allows plants to anticipate upcoming light transitions and to predict duration of the current and future alternating light and dark periods. Plants can also predict the change of seasons and find the optimal time for their flowering and fruit bearing.

The daily alternate periods of light and darkness induce diurnal rhythms that are visible in all major physiological processes and responses of plants [6,7]. They allow metabolic processes in plants to be phased and occur at a specific time of the day [8]. Microarray studies on the *Arabidopsis* genome have shown daily rhythmicity to appear in the expression of some 6–16% of all genes [9], resulting in coordinated timing of daily events [10].

Timing in plants is maintained by the circadian clock (oscillator), an endogenous oscillator present in every cell of the plant body. The circadian clock is not a discrete cellular structure, but the consequence of interconnected transcriptional and translational feedback loops in the expression of genes that form the core of circadian clock [11]. Functionally, the circadian clock consists of three main components: input, core genes, and output [12]. Input refers to receptors that can perceive light signals [13]. Light receptors comprise mainly the phytochromes and chryptochromes [14,15], with participation of other pigments such as zeitlupe [16]. Output genes transmit the functional states of the clock oscillator to metabolic or developmental processes. Hypocotyl elongation is a known output of the circadian clock, and it is often used in experimental studies [17] to monitor clock function in real time.

Pigments involved in light inhibition include phytochromes and chryptochromes [18,19,20,21], although phototropins [22,23] and other pigments may also contribute. Signal transduction pathways starting from individual pigments and their crosstalk have also been extensively studied [24,25], especially with the use genome-wide surveys (microarrays) detecting large numbers of signaling components [26]. Some components such as SPA1 even showed a promoting effect on hypocotyl elongation and counteracted the growth inhibition mediated by phyA and phyB [27].

However, the major factor determining whether plantlets will follow photomorphogenesis or skotomorphogenesis is the activity of the COP9 signalosome (CSN), a protein complex supposed to function as the main light switch in plants [28], involved in the regulation of a number of developmental processes. Triggered by the presence of light, it acts as a negative regulator of photomorphogenesis [29], preventing plants from starting de-etiolation in darkness. The presence of light also affects the metabolism and activity of most phytohormone groups [30], as in the case of gibberellins, which also represses photomorphogenesis in darkness.

Phytochromes and cryptochromes are known to be the key pigments in the light entrainment of circadian rhythms [13,14]. Entrainment enables plants to anticipate timing of imminent light transitions and duration of daytime and nighttime periods of the current photoperiod.

Sunflower was a popular model system in early developmental and tropism studies of dicotyledonous plants [31,32,33]. Studies of hypocotyl elongation [34,35,36,37] failed to detect rhythmicity of hypocotyl elongation in plants grown under diurnal photoperiods. This was probably caused by the use of a small number of daily measurements. Digital imaging and related techniques simplified and gave a new impetus to studies of plant growth and development [38]. The use of digital imaging techniques allowed accurate studies of sunflower seedling circumnutation and showed that hypocotyl elongation has a distinct diurnal rhythmicity [39].

The action spectra determined for inhibition of hypocotyl elongation in *Raphanus sativa* [40] and *Sinapis alba* [41] showed that blue, red, and far-red light components were the most inhibitory in dark-grown plants, as shown previously for *Cucumis sativus* [42]. The involvement of two different light receptors in the control of seedling growth has been a long-standing concept, although the relative importance and contribution of the receptors appears to vary between different species [43].

Controversial effects of blue light in hypocotyl elongation have been reported in several plant species, although blue light clearly exerts an inhibitory effect in most of them, as in *Arabidopsis*. In potato shoot cultures, it was found that removal of blue light from white light using yellow filters significantly promotes shoot elongation [44]. However, the situation is different in sunflower, where blue light has been reported to stimulate hypocotyl elongation [33,45]. On the other hand, in dark-grown sunflowers, blue light was shown to induce a strong but transient suppression of hypocotyl elongation [46]. Similar observations of transient suppression in hypocotyl elongation in *Arabidopsis* have also been reported [22], indicating that phototropin is responsible for this rapid, initial growth inhibition. In *Arabidopsis,* all wavelengths except green [47] were found to inhibit hypocotyl elongation starting at low fluence values [25].

We investigated rhythmic hypocotyl elongation in diurnal photoperiods with artificial white light provided as daytime lasting 8, 10, 12, 14, or 16 h, comparing it with the uniform elongation characteristic for free-running LL and DD conditions. The study was extended to cover effects of monochromatic blue, green, yellow, and red light applied in various free-running, diurnal, or other light combinations. Illumination of plants with a single monochromatic light supported only uniform elongation, abolishing rhythmicity to the same extent as the extended duration of daytime or nighttime periods in plants entrained to diurnal photoperiods. In darkness, hypocotyl elongation was variable, depending on the spectral composition of light to which plants were exposed prior to the start of darkness. Our data indicate that red light has a suppressive effect on hypocotyl elongation but only when it is applied in combination with other monochromatic light colors. White light, being a mixture of many different light colors including red, is highly suppressive to hypocotyl elongation, extending this suppressive effect to last during the period of darkness that follows the daytime in diurnal photoperiods. Therefore, hypocotyl elongation in young sunflower seedlings results from the balance and interaction of light components that can support or suppress hypocotyl elongation as suggested by Parks et al. (2001) [27]. We also briefly discuss how the spectral composition of light, as well as its periodicity, affects the establishment and maintenance of light entrainment.

## 2. Material and Method

### 2.1. Plant Material and Germination

**Figure 1:**
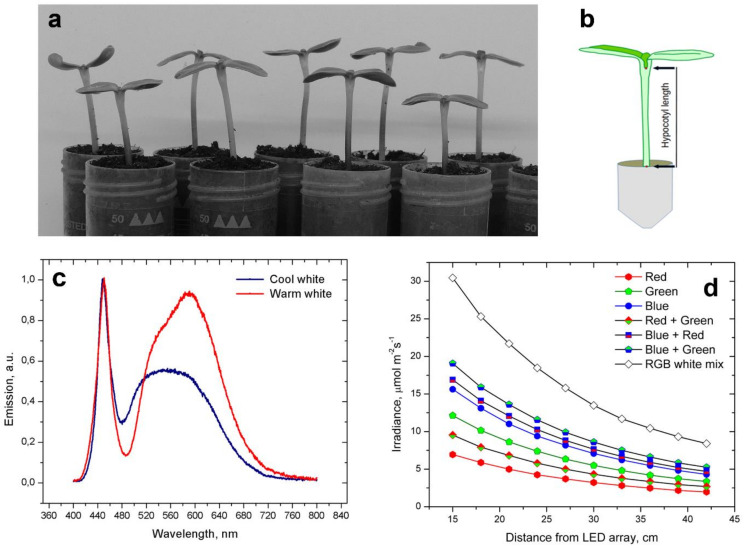
Plant material, measurements of hypocotyl length, and spectral light characteristics of some light sources. (**a**) Plants growing in a diurnal 14/10 h light/dark photoperiod on the sixth day from the onset of germination. (**b**) Schematic diagram of a plantlet showing how relative hypocotyl length was measured. (**c**) Emission spectra of cool-white and warm-white LED panels measured with the Ocean UV NIR 2000 spectrophotometer. Both LED panels had a low emission of far-red light above 700 nm. (**d**) Irradiance in various single, dual, or triple monochromatic light waveband combinations produced by a densely packed array of RGB LED strips (V-Tac, SKU 2124). Irradiance measured with LiCor 250 with a quantum sensor.

Seeds of cv. Kondi (Syngenta, ChemChina, Basel, Switzerland) were washed in water for 1 h (imbibition) and then placed in PVC boxes under layers of moist paper towels at 24–25 °C for germination. Germination occurred in dim light or darkness. Some 24–36 h after the start of imbibition, the seeds were inspected for germination success. Those whose radicles had reached 3–5 mm in length were sown in 50 mL PVC centrifuge tubes filed to the rim with peat-based potting mixture (Figure 1a). The plantlets in tubes were well watered during sowing, and no additional watering was required until the end of treatments. Tubes with the sown plantlets arranged in Styrofoam trays were placed in growth chambers with a suitable photoperiod. The temperature was maintained at 24–25 °C.

### 2.2. Light Sources

For diurnal photoperiods with white light, Philips TLD fluorescent lamps or 30 × 30 cm LED plates were used, both providing irradiance of 70 µmol·m^−2·^s^−1^. Hypocotyl elongation was studied in diurnal photoperiods of 8/16, 10/14, 12/12, 14/10, and 16/8 h of light/darkness (LD), which were designated as short, moderately short, neutral, moderately long, and long day photoperiods, respectively, according to their day length.

Free-running conditions included constant white light (LL), constant darkness (DD), and constant monochromatic blue light (BB at 470 nm), green light (GG at 560 nm), yellow light (YY at 600 nm), or red light (RR at 660 nm).

Monochromatic light was also tested in diurnal 14/10 h LD photoperiods in which monochromatic light was applied as daytime, in 4 h long T-cycles alternating with darkness, or in other combinations.

Monochromatic light was produced by LED lights sources such as Philips GU10 spot lamps, V-Tac LED strips, and LED arrays with high irradiance from unknown manufacturers. Emission spectra of various LED light sources were measured using an Ocean NIR UV 2000 spectrophotometer. White LED panels had a nearly continuous emission of visible light rich in blue, yellow, and orange portions of the spectrum (Figure 1c). Irradiance was measured using Li-Cor 250A light meter with quantum sensor (Figure 1d).

The time of imbibition was considered as the time of dawn for diurnal or subjective dawn for free-running photoperiods. For imagining during the nighttime darkness, plants were briefly (seconds) illuminated at 2 h intervals with green or yellow LED light at an irradiance of 0.45 µmol·m^−2^·s^−1^ or less. A longer duration of irradiance could affect the hypocotyl elongation pattern.

### 2.3. Treatments and Imaging

Batches of 15–20 tubes containing germinating plantlets were arranged in Styrofoam trays in front of the camera. For each treatment, the batches were replicated three times or more. The curves in the figures are averages of plants in a representative treatment batch.

Plantlets were oriented so that their cotyledonary axis was transverse to the imaging camera. Images were captured using the time-lapse function of Nikon P520 and P510 cameras at 10 min intervals. Arrangement of tubes with plantlets enabled good frontal visibility of the entire hypocotyl with the cotyledon petiole junction as the external marker of hypocotyl length (Figure 1b). The suture between the cotyledons was visible as soon as the protective hulls slipped from the expanding cotyledons. The thick, black-colored hulls of cv. Kondi achenes seemed to be impenetrable to light, preventing early light priming.

The start of imaging was connected with the developmental stage of plantlets, requiring them to be at the advanced stage of hook straightening. In most treatments employing monochromatic light, the start of imaging was significantly delayed. The time available for imaging also varied between treatments depending on their elongation rates. Those with lower elongation rates as in diurnal photoperiods could be followed much longer than those in treatments with monochromatic light that induced fast hypocotyl elongation.

### 2.4. Hypocotyl Length Measurements

The length of the hypocotyl was measured with Jstore software using digital images, measuring the distance between the tube rim and the cotyledon petiole junction position, as presented in Figure 1b. The hypocotyl length registered in this way was the relative hypocotyl length, as the actual hypocotyl length could only be estimated.

The relative hypocotyl length presented in the figures is the cumulative increase in hypocotyl length. The hypocotyl elongation rate was calculated and provided in 2 h increments, while the overall elongation rate refers to the elongation rate of the longer periods of time or to the entire treatment duration.

All key findings obtained with cv. Kondi were confirmed in treatments with the early-bearing sunflower hybrid NS H7749, which shows rapid development of plantlets.

## 3. Results

### 3.1. Rhythmic Hypocotyl Elongation in Diurnal White Light/Dark Photoperiods

**Figure 2:**
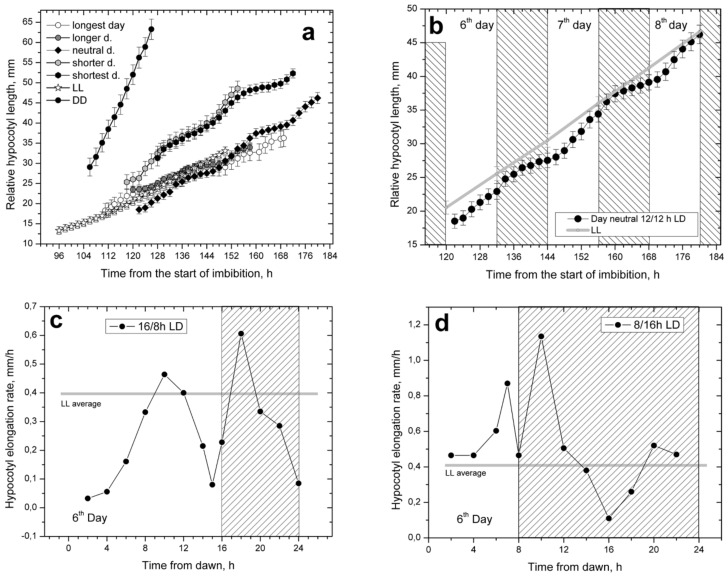
Hypocotyl elongation in diurnal light/dark (LD) photoperiods with light of white LED panels for daytime and true dark for nighttime. (**a**) Five LD photoperiods with rhythmic elongation had similar overall elongation as the free-running constant white light (LL) treatment with uniform hypocotyl elongation. Elongation in the free-running darkness (DD) was much higher than in any of the photoperiods with white light, (**b**) Hypocotyl elongations in day neutral 12/12 h LD photoperiod and LL were highly compatible. Hypocotyl elongation rates in long day 16/8 h LD photoperiod (**c**) and short day 8/16 h LD photoperiod (**d**).

In the five diurnal photoperiods that were studied, hypocotyl elongation showed a prominent daily rhythmicity, resulting in characteristic stairway-shaped elongation patterns visible in curves obtained by plotting the cumulative increase in hypocotyl lengths (Figure 2a). The overall elongation rates were similar in all five photoperiods, although the photoperiods with short and moderately short days showed a slightly faster initial hypocotyl elongation than the photoperiods with long and moderately long days. They could be grouped together with the photoperiod of neutral day 12/12 h LD, which occupied the central position. The overall elongation rate of the rhythmic elongation in the neutral day photoperiod was similar to the uniform elongation of plants grown under LL conditions (Figure 2b). Therefore, all photoperiods that contained white light could be placed together in the same elongation rate group, regardless of their rhythmicity.

The rhythmicity of the diurnal photoperiods was best observed and analyzed in graphs in which changes of hypocotyl elongation rates were plotted over time (Figure 2c,d). The graphs showed two daily elongation peaks separated by periods of decreased (arrested) elongation. There was a daytime peak around midday or slightly later and then another peak in the nighttime, 1–2 h after dusk. Two daily periods of low elongation (daily arrests) with minimal elongation values were located at the beginning and at the end of the daytime period. The positions of the two daily peaks and the minimum at the end of daytime were fixed, while the position of the second minimum was variable depending on the length of nighttime.

The dual nature of the hypocotyl elongation kinetics which differed between daytime and nighttime periods was prominent in the long and moderately long day photoperiods. In the short and moderately short photoperiods, the dual nature of hypocotyl elongation was less pronounced. The time between the two elongation peaks became shorter as the daytime duration decreased, as seen for the arrest at the end of day (Figure 2d). Thus, in photoperiods with short and moderately short days, hypocotyl elongation was dominated by the long-lasting nighttime with asymmetric position of its elongation peak. Interestingly, the nighttime elongation minimum was located 8 h after dusk in both the short-day and the long-day photoperiods, suggesting that it may be a pivotal, nighttime recovery point. A similar nighttime recovery point was previously described for the phototropic bending ability of sunflower seedlings [44].

### 3.2. Uniform (Arrhythmic) Elongation in Free-Running White Light (LL), Darkness (DD), or Monochromatic Light

**Figure 3:**
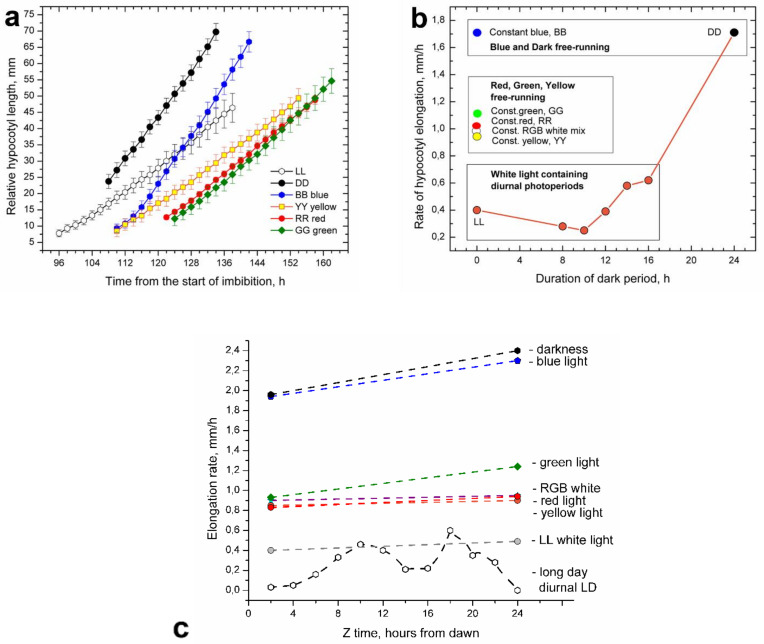

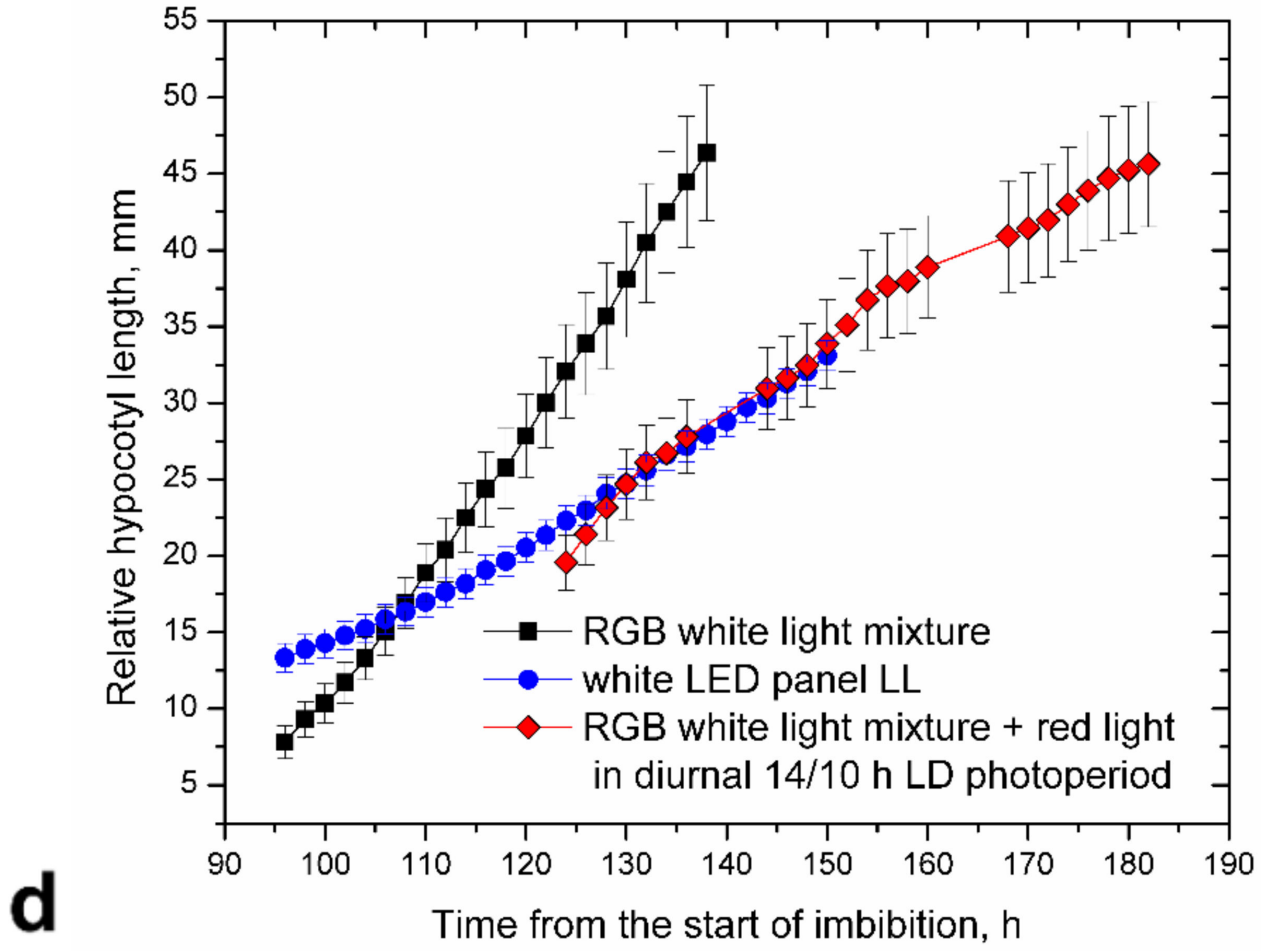
Hypocotyl elongation in plantlets grown in free-running monochromatic light is rapid and uniform. (**a**) Cumulative hypocotyl length increase under the green (GG), red (RR), yellow (YY), and LL, i.e., an RGB mixture of blue, red and green light. The elongation rates were similar but treatments differed with respect to the time at which the hypocotyls began to elongate. Elongation under the blue light (BB) was much faster and could be grouped with elongation in true darkness (DD). (**b**) Comparison of hypocotyl elongation rates in diurnal (white-light LED panels + dark) photoperiods and free-running monochromatic light treatments with respect to the daily duration of the true nighttime (darkness) period. For free-running monochromatic light treatments, the duration of darkness was zero. (**c**) Elongation rates of plants grown under various monochromatic light colors compared to rate in diurnal moderately long day photoperiod. Elongation covered 24 h of the sixth day from the onset of germination. In monochromatic light, there was only a uniform type of hypocotyl elongation. (**d**) Hypocotyl elongation in LL conditions provided by light of white LED panels, with a radically different “white” RGB LED light mixture. When plants in the RGB LED light mixture were supplemented with additional 20 µmol·m^−2^·s^−1^ of red light, their elongation rate decreased to the elongation observed in plants under white LED panels. Suppressed elongation was also visible in the interpolated periods of darkness.

Under the free-running LL and DD conditions, hypocotyl elongation was uniform (arrhythmic), but it gradually increased over time (Figure 3a). Elongation at DD was significantly faster than at LL. Plants grown in DD were etiolated, with long hypocotyls and undeveloped cotyledons, still partially enclosed by seedling husks. LL plants were dark green, husk-free, well developed, and vigorous in appearance. Plants from LL and those from diurnal LD photoperiods had similar overall elongation rates; thus, they could be grouped together (Figure 3b).

Etiolated plantlets germinating under the DD conditions emerged above the soil with typical hooks that gradually straightened. However, cotyledon opening and subsequent cotyledon expansion in plants from DD were delayed. Vigorous hypocotyl elongation was uniform, but it accelerated somewhat over time (Figure 3a). Etiolated DD plants placed back in LL or diurnal photoperiods continued their very fast elongation during the first 8 h, showing that fast elongation induced by DD could not be quickly suppressed by white light.

In free-running conditions equipped with monochromatic LED light, hypocotyl elongation was also uniform, as seen in LL and DD (Figure 3a). Hypocotyl elongation in blue (BB) light was nearly high as in DD, albeit with a somewhat delayed start. Green (GG), yellow (YY), or red (RR) light provided mutually similar and parallel graphic almost identical to the elongation in the RGB white light mixture. Elongation of this group was delayed and slower than in the DD and BB elongation group, but still much faster than in the group containing LL and diurnal LD photoperiods (Figure 3b). Thus, elongation under blue monochromatic light (1.9 mm/h) was nearly five times faster than the overall elongation rate in the diurnal moderately long day photoperiod (0.4 mm/h) Figure 3c. Elongation rates of plants during the sixth day after the onset of germination, growing under different monochromatic light colors and diurnal moderately long day photoperiod, are presented in Figure 3c.

The differences in elongation rates between DD and monochromatic lights were less striking than the large delays observed in their start of elongation. In contrast to monochromatic lights, elongation in LL treatment was not delayed, and it began at the same time as in DD conditions.

RGB LED strips with all three colors turned on produced a bluish-green mixture of white light, providing equally high hypocotyl elongation as in RR, GG, and YY (Figure 3b). However, elongation rates of plants growing under the RGB triple white-light mixture and those of plants under white light (LL) were significantly different. Supplementing more red light to the RGB white-light mixture suppressed this excessive hypocotyl elongation even if dark periods were interpolated (Figure 3d).

Elongation of the hypocotyl under monochromatic light in free-running conditions was not dependent on irradiance levels (data not presented). Elongation responses were, therefore, saturated at very low light intensities. Thus, even a 10-fold change in irradiance levels resulted in little or no change in the elongation rate of plants grown under blue or red light. Only in the case of blue light at the beginning of irradiation was there a transient decrease in hypocotyl elongation lasting up to 2 h, which was visible in all experimental treatments.

Blue light was the only component of visible light that could trigger the phototropic (PT) bending in sunflower seedlings, strongly suppressing circumnutational movements. Employing the conditions elaborated previously [48], we showed that PT bending was also saturated at very low blue light irradiance, as in the case of hypocotyl elongation. Vigorous PT bending was observed under continuous blue light at irradiation as low as 0.5 µmol·m^−2^·s^−1^(data not presented).

### 3.3. Irradiation with Two Different Colors of Light

**Figure 4:**
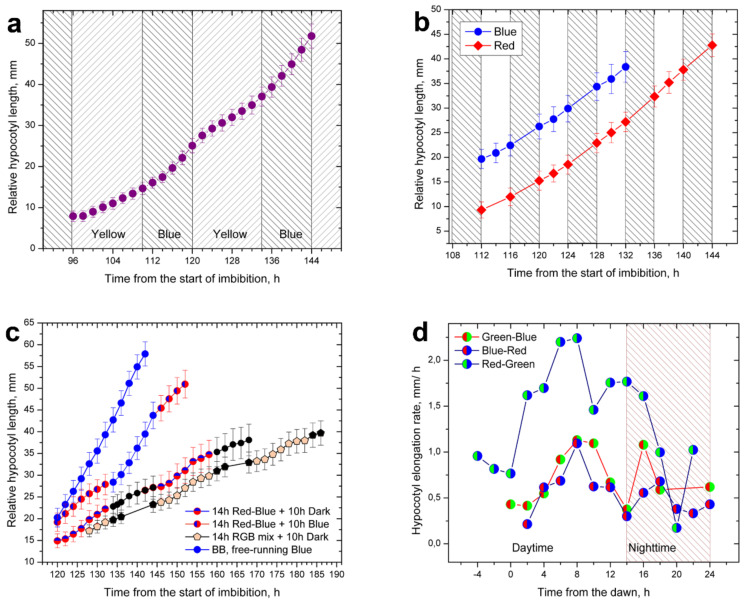
Alternating and simultaneous application of monochromatic light. (**a**) Hypocotyl elongation in a photoperiod in which 14 h long periods of yellow light alternated with 10 h long periods of blue light. In each of the periods, non-suppressive hypocotyl elongation was an independent event that followed its own rules. (**b**) Four hour long T-cycles in which red or blue light alternated with darkness, which was not suppressed by previous blue or red light illumination. Blue periods following darkness had low initial elongation rates that later improved. (**c**) Hypocotyl elongation in treatments with a suppressive red + blue dual illumination followed by true darkness or blue light. Suppressive red + blue light continued to suppress elongation in the following period of true darkness but not in the blue light. Therefore, darkness remained suppressive after the red + blue doublet, whereas blue light promoted hypocotyl elongation after red + blue. (**d**) Hypocotyl elongation in plants illuminated for 14 h with three distinct RGB doublets followed by 10 h periods of darkness. High hypocotyl elongation was promoted only in the blue + green doublet, which lacked the early night peak found in white light diurnal photoperiods.

In treatments in which monochromatic light alternated with darkness or in those where two different monochromatic lights alternated, hypocotyl elongation was uniform with rates characteristic for the currently employed light colors (Figure 4a,b). Changes in the elongation rates at the end and at the beginning of a new period were abrupt.

Simultaneous illumination of seedlings with two different monochromatic light colors (light doublets) during a 14 h long daytime followed by a 10 h long dark period resulted in gross changes in the hypocotyl elongation patterns. Uniform hypocotyl elongation was abolished and replaced by complex responses including those resembling rhythmic elongation in diurnal photoperiods provided with white light (Figure 4c).

In the blue–green color doublet, hypocotyl elongation was very high with a strong peak in the 14 h long “daytime” period. The peak in the nighttime was either absent or postponed. In the other two doublets (red–green and red–blue), hypocotyl elongation was much lower but there were two noticeable daily peaks. One peak was in the middle of the “daytime” and the other in the nighttime, just as in the white light diurnal photoperiods.

The absence or delay of the nighttime peak in the blue–green doublet suggests that the absence of nighttime peak may have been caused by the presence of red light in the “daytime” of other doublets. Although red light was provided at rather low irradiance (Figure 1b), blue and green light could not cancel the suppressive effect of red light when they were present together in doublet combinations. Similarly, green light could not prevent hypocotyl elongation when present in doublet together with blue light. The treatment with red–green light was the doublet that best restored the diurnal rhythmicity.

We also exposed the plants to alternating periods of single and dual light irradiance. This was achieved by providing 14 h long periods of red light interpolated into a background of a free-running blue light treatment. In this way, a diurnal photoperiod (RB+B) was created with a 14 h “daytime” provided by the red–blue doublet, followed by a 10 h blue light “nighttime” (Figure 4d). Blue light was actually free-running, being present at the same irradiance level in both light periods. During the red–blue “daytime” (120–128 h), hypocotyl elongation was suppressed in the same way as during the daytime of white-light diurnal LD photoperiods. In the blue nighttime that followed for the next 10 h, plantlets slipped into very fast uniform type elongation characteristic for BB treatments. The blue-light nighttime strongly influenced the next red–blue daytime period, which started at 144 h and also appeared uniform. A control treatment (RB+D) which also had a 14 h long red–blue doublet for daytime but followed by a true dark nighttime period, showed a completely different response as elongation was suppressed in true darkness and not stimulated as in the blue nighttime of RB+B. The control RB+D treatment was similar to another treatment (RGB+D) that used an RGB white-light mixture for daytime followed by a 10 h long dark nighttime. The RB+D and RGB+D treatments differed only by the presence of green light in the RGB mixture, which resulted in no significant differences in their elongation patterns.

Dark treatments resulted in rapid elongation of seedlings that were not previously illuminated. When a dark period followed illumination with red light, then elongation was suppressed. Blue light can overcome suppression induced by red light, providing fast elongation during the blue nighttime period. It remains to be tested how long this red light-induced suppression can last.

This experiment showed that red light (alone or in presence of other light colors) was the factor suppressing hypocotyl elongation. It was also responsible for the establishment of diurnal rhythmicity as hypocotyl expression is suppressed at nighttime with true darkness. Blue light applied at nighttime instead of true darkness overcame the red light-induced suppression, enabling plantlets to slip into the uniform pattern of hypocotyl elongation.

Blue light only superficially resembled darkness, supporting a high rate of hypocotyl elongation. Plantlets in blue light were elongated but not etiolated, and they suppressed circumnutations that otherwise appeared in true darkness.

### 3.4. Entrainment in LD Photoperiods and Maintenance of Rhythmicity

The pattern of hypocotyl elongation of all treatments whether rhythmic or uniform was evident at dawn of the sixth day, 120 h after the onset of germination. The time available to plantlets from diurnal photoperiods to establish light entrainment was very short. The first signs of successful germination, visible as local elevation of the soil surface, were observed about 72–96 h after the onset of imbibition. By the fifth day, 96 to 120 h after the onset of germination, the plumule was at least partly exposed to direct light at the soil surface on the fifth day. Establishment of light entrainment was, therefore, limited to a short period of time, barely exceeding a day.

The presence of entrainment in LD diurnal photoperiods was recognized by elongation rates that changed significantly throughout the day, with peaks and periods of arrested elongation. Successful entrainment enables plantlets to position the peak of daytime hypocotyl elongation at midday and to anticipate the onset of the transition from light to dark at dusk. In other words, plants anticipate the position of daily elongation maximum before the day is over. Thus, entrainment appeared to do nothing more than repeat the situation established the previous day. In the nighttime, the elongation peak was always located 2 h after dusk and the beginning of the dark period. It is questionable whether the plants anticipated the end of night period or simply adjusted to the start of new day when it came. Hence, what happens if dawn simply fails to appear?

**Figure 5:**
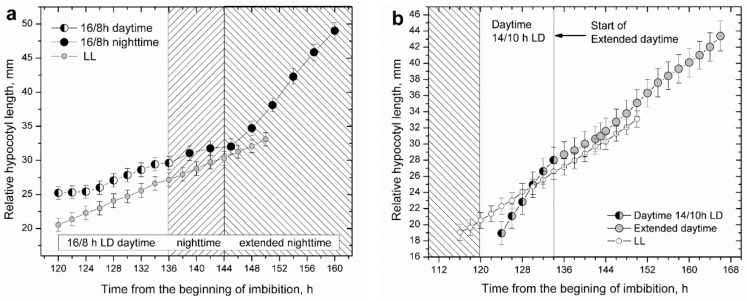
Rhythmic hypocotyl elongation of diurnal photoperiods rapidly changes to uniform (arrhythmic) elongation when the anticipated duration of the daytime or nighttime is breached. This happens when daytime or nighttime duration is extended. (**a**) Prolonged nighttime duration. At the expected end of nighttime, the lights were not turned on and there was no dawn. Shortly after the onset of extended darkness, hypocotyl elongation accelerated considerably and became uniform. (**b**) Prolonged daytime duration. At the expected end of daytime period, the light was not turned off and there was no dusk. In the extended daytime, elongation became uniform and the expected nighttime peak 1–2 h from dusk was absent. After the first 10 h of the prolonged daytime, which corresponds to the subjective night, hypocotyl elongation showed a small, transient increase. the extended daytime and nighttime durations both appeared as de novo start of free-running LL or DD conditions.

To solve this dilemma, we designed treatments in which the last day or last night of plantlets well entrained to their 14/10 h LD diurnal photoperiod were unexpectedly extended, mimicking de novo start of a free-running LL or DD condition (Figure 5a,b).

In both cases, the circadian clock did not cut in as expected for a functional circadian regulation, and plants failed to manifest the elongation pattern characteristic for the anticipated light period. After just 1–2 h spent in a regime of extended daytime or nighttime plants switched from diurnal rhythmic to uniform, free-running elongation patterns: LL for extended daytime and DD for extended nighttime.

Apparently, the circadian clock malfunctioned as plants entered a uniform elongation pattern in which the circadian clock functionality seemed to be absent, nonvalid, masked, or simply overridden. In the case of extended nighttime, the highly accelerated elongation rapidly brought the hypocotyl length to high values that prevented further measurements. In the case of extended daytime treatments, the outcome of daytime extension could be followed much longer. During the first 10 h of growth in the extended daytime, corresponding to the subjective nighttime, hypocotyl elongation was uniform, and the peak corresponding to the expected nighttime maximum was absent, appearing as a failure of circadian regulation. However, in the continued duration of the extended day, corresponding to the subjective day, some circadian regulation reappeared, such that, in the following subjective daytime period, weak hypocotyl elongation could still be observed according to the diurnal elongation pattern. This means that circadian regulation was present and functional, but strongly suppressed or overridden.

Therefore, an answer emerges indicating that, in sunflower seedlings, circadian regulation may be suppressed in some situations and overridden by other regulatory mechanisms. In our concurrent study dealing with sunflower phytohormones and their circadian rhythmicity [49], we will show correlations among light duration, light transitions, and phytohormone production, demonstrating that circadian regulation goes on in LL conditions with uniformity of responses due to the synchronization of genes associated with the core of the circadian clock.

## 4. Discussion

We studied the development of sunflower seedlings under various white-light sources such as fluorescent lamps, LED panels, or LED strips. At an irradiance of 70 µmol·m^−2^·s^−1^, white light suppressed the hypocotyl elongation irrespective of the light source, as seen under natural conditions. In diurnal photoperiods, this suppression persisted throughout the dark period (nighttime period) of entrained plantlets. The dark periods (nighttime) were of utmost importance in maintaining diurnal rhythmicity and sustained suppression of hypocotyl elongation.

### 4.1. Suppression and Promotion of Hypocotyl Elongation

White light suppressed hypocotyl elongation irrespective of its periodicity, showing the same suppressive effect both in diurnal and in LL conditions. Under diurnal photoperiods, hypocotyl elongation actually depended on the duration of nighttime (Figure 3b), similar to *Arabidopsis*, in which hypocotyl length was found to increase with increased nighttime duration of photoperiods [50] (Figure 1a). Sunflower plantlets apparently compensated for the large difference in daytime duration of diurnal photoperiods.

The light of monochromatic LEDs could not suppress hypocotyl elongation; after numerous attempts, we had to give up and admit that monochromatic light in sunflower has a promotive effect on hypocotyl elongation. In a situation in which light exerted both suppressive and stimulatory effects, we had to carefully dissect the effects of individual wavelengths and their combinations. The first task was to establish a growth rate limit that separated suppressive from stimulative light effects. In photoperiods with white light, elongation rates were lower than 0.6 mm/h and considered as suppressive, since, in monochromatic light, elongation rates were 0.8 mm/h or higher, and they were considered as stimulative (Figure 3b).

The stimulatory effect of monochromatic light was not related to irradiance, as it was recorded both in the lower (1–5 µmol·m^−2^·s^−1^) and higher (40–50 µmol·m^−2^·s^−1^) irradiance levels of blue and red light. Even large changes in the irradiance during the same treatment were hardly detected in the graphs of hypocotyl elongation. Obviously, the stimulative effect of monochromatic light on hypocotyl elongation had a rather low saturation value. Therefore, monochromatic safe lights with long exposure time cannot be recommended for use in manipulations with plants during the nighttime.

Data from our experiments confront the classic concept of white-light diurnal photoperiods in which suppression of hypocotyl elongation by white light is extended during the nighttime. Monochromatic light is not suppressive per se, and dark periods that follow illumination with monochromatic light are also not suppressive, as can be seen in the 4 h T-cycles in Figure 4b, or when longer dark periods alternated with monochromatic light, as in case of blue and yellow in Figure 4a. Thus, a modified diurnal photoperiod, in which monochromatic light alternates with true darkness, cannot suppress hypocotyl elongation nor can it provide entrainment.

Dual illumination with two different monochromatic lights applied together restored elongation suppression in doublet combinations containing red light. Treatments in which blue and green light were applied together strongly stimulated hypocotyl elongation and promoted only a “daytime” elongation peak, as shown in Figure 4d. The data confirm the well-known concept that multiple pigment systems are involved and operational in the reception and transduction of light signals in plants. This was suggested from the earliest studies and confirmed later as the transduction pathways for light signals became better characterized [51,52]. However, there seems to be no point in comparing *Arabidopsis* and sunflower when their light requirements and light responses are so different. The components are the same, homologous, or compatible, but their arrangement and functioning in *Arabidopsis* significantly differ in comparison to sunflower [53]. In the case of sunflower, background knowledge of the pigments involved in photomorphogenesis is still sparse, so we are forced to discuss light effects in terms of their absorption wavelengths rather than the actual pigments transducing the light signals. This is a task for future studies.

Interestingly the white-light mixture produced by RGB 5050 LED strips stimulated hypocotyl elongation, providing equally high elongation rates to monochromatic red or green light (Figure 3b). However, red light in this mix was fairly low (Figure 1d), and providing additional red light to plants at 20 µmol·m^−2^·s^−1^ together with the RGB mixture efficiently suppressed hypocotyl elongation.

### 4.2. Antagonistic Effects of Monochromatic Light

We confirmed that blue light has both a suppressive and a promotive effect on hypocotyl elongation. Suppression is visible as the initial effect, expressed only at the start of blue-light illumination, gradually changing into significant promotion, resulting in overall elongation rates equivalent to those of dark-grown etiolated plants. Therefore, both reports showing a suppressive [46] and promotive effect of blue light [45] are valid.

Red light was promotive when applied alone, but suppressive when combined with other wavelengths as in white light. Darkness that followed monochromatic red light was promotive, as seen in 4 h long red/dark cycles (Figure 4b). However, when darkness followed treatments with dual red + another light color or light from white LED plates, then hypocotyl elongation was suppressed, indicating that it is a prolonged state which needs time to expire. This expiring time seems to be stored by entrainment.

The use of mixed single and dual monochromatic light illumination gave some interesting responses, as shown in Figure 4c. Suppression of hypocotyl elongation induced by the combination of red and blue illumination continued in the dark, but not in the “nighttime” with blue light. The blue nighttime light treatments could also be interpreted as free-running blue light treatments with superimposed periods of red light, which caused temporary suppression of hypocotyl elongation. Thus, it could also be interpreted as free-running blue light (BB) with suppressive red light occurring only during the “daytime”. Being continuous, the initial blue light decline in hypocotyl elongation occurred only once at baseline.

We did not examine far-red light as it is mostly absent from light produced by white-light LED panels, especially in the cool white type (Figure 1b). However, it is known that far-red light applied alone stimulates elongation of sunflower shoots and hypocotyls [33,54]. Far-red light applied together (in a doublet) with red light leads to responses that depend on the mutual ratio of the two red colors. Thus, a higher proportion of red light (low red/far-red ratio) had an inhibitory effect, while a higher proportion of far-red light (high red/far-red ratio) stimulated elongation of sunflower internodes and hypocotyls [53]. Interactions of red and far-red light are known to be involved in the plant shade avoidance responses [55].

### 4.3. Entrainment and Circadian Regulation

The slippage of the elongation pattern from rhythmic to uniform (arhythmic), when the expected duration of nighttime or daytime periods was exceeded, was initially considered to be a malfunction of circadian regulation and its associated entrainment. However, we then observed the same response when plants were exposed to monochromatic light. In spite of this new finding, the relation between the rhythmic and uniform elongation patterns became more complex. These new findings show that the changes in elongation pattern can be triggered rapidly at the very beginning of signal transduction lines, suggesting that different regulatory mechanisms are involved and not just circadian regulation.

Our results suggest that entrainment is dependent on the spectral composition of light provided to plants. At present, it seems that the presence of red light appears to be a mandatory requirement for light entrainment, at least in cv. Kondi, but such a conclusion requires more evidence from future studies spanning different sunflower genotypes.

## 5. Conclusions

There are two different situations in which light entrainment in sunflower is abolished and hypocotyl elongation changes from rhythmic to a uniform pattern during diurnal photoperiods. The first case is associated with missed anticipation timing and appears to be distinct from the later discovered failure of entrainment, which is triggered simply by illumination with monochromatic light. While the first case may be related to failure of the circadian clock to regulate metabolic pathways, the second case appears to be related to specific light receptors and interactions between their signal transduction pathways.

To understand the data that we collected, it was necessary to accept a new attitude in which light is not considered as a purely inhibitory factor of hypocotyl elongation. Instead, light should be considered as a factor with both suppressive and promotive effects. Our findings, therefore, support the view put forward by Parks et al. (2001b) stating that both stimulative and suppressive light factors are present and balanced in the regulation of hypocotyl elongation [27]. Conversely, elongation in darkness can be either suppressive or enhancing, depending on the light conditions provided to the plants before the onset of darkness.

Various treatments that have promotive and suppressive light effects on hypocotyl elongation are summarized in Table 1.

Data that we presented here are far from final. They cover the main findings, indicating the direction in which studies should be continued. Elucidating the effects of monochromatic light combinations in regulating hypocotyl elongation and establishment of light entrainment needs to be extended to cover diverse sunflower genotypes.

## Figures and Tables

**Table 1:** Overview of the light quality (waveband composition) used in the treatments, their irradiance levels, and their effects on hypocotyl elongation.

Light Treatment	Irradiance	Effect on Hypocotyl Elongation
True darkness from the start of germination	none	Highly promoted
Darkness in diurnal photoperiods with white light daytime	none	Suppressed
White light (LED plates, strips, and fluorescent lamps)	irradiance up to 70 µmol m^−2^ s^−1^	Suppressed
White-light mix of RGB LED strips	20 µmol m^−^^2^ s^−^^1^	Promoted
White-light mix of RGB LED strips supplemented with 20 µmol·m^−2^·s^−1^ of red	40 µmol m^−^^2^ s^−^^1^	Suppressed
Blue monochromatic	1–50 µmol m^−^^2^ s^−^^1^	Highly promoted
Green monochromatic	2–20 µmol m^−^^2^ s^−^^1^	Promoted
Red monochromatic	4–60 µmol m^−^^2^ s^−^^1^	Promoted
Yellow monochromatic	4–20 µmol m^−^^2^ s^−^^1^	Promoted
Red + blue dual	44 µmol m^−^^2^ s^−^^1^	Suppressed
Red + green dual	45 µmol m^−^^2^ s^−^^1^	Suppressed
Blue + green dual	35 µmol m^−^^2^ s^−^^1^	Promoted
Blue after red–blue dual	20 µmol m^−^^2^ s^−^^1^	Promoted
Darkness after red–blue dual	none	Suppressed

## Data Availability

Data presented in this study are available from the authors.
